# Impact of transcutaneous electrical acupoint stimulation on postoperative recovery in older adults following pterygium excision: a prospective randomized controlled trial

**DOI:** 10.3389/fneur.2025.1631005

**Published:** 2025-10-09

**Authors:** Xinlu Wu, Ledan Huang, Huanhuan Sun, Yating Chen, Haibo Guo, Yuchen Wang, Jingye Pan, Xinxin Wang, Yunchang Mo, Qinxue Dai

**Affiliations:** ^1^Department of Anesthesiology, The First Affiliated Hospital of Wenzhou Medical University, Wenzhou, Zhejiang, China; ^2^Department of Intensive Care Unit, The First Affiliated Hospital of Wenzhou Medical University, Wenzhou, Zhejiang, China; ^3^Department of Operation Room, Wenzhou Hospital of Integrated Traditional Chinese and Western Medicine, Wenzhou, Zhejiang, China

**Keywords:** enhanced recovery after surgery, postoperative anxiety, postoperative pain, transcutaneous electrical acupoint stimulation, pterygium

## Abstract

**Objective:**

The aim of this study is to evaluate the effect of transcutaneous electrical acupoint stimulation (TEAS) during the perioperative period on the quality of postoperative recovery among patients undergoing pterygium excision.

**Methods:**

A total of 110 patients scheduled for unilateral pterygium excision were enrolled and randomly assigned in equal numbers to the TEAS group or the control group. In the TEAS group, patients received TEAS at the LI4 and PC6 acupoints, initiated 30 min before anesthesia induction and continued until the conclusion of surgery. In the control group, patients had electrode pads applied without active stimulation. Numerical Rating Scale (NRS) scores, State–Trait Anxiety Inventory (S-TAI) scores, and Quality of Recovery-40 Questionnaire (QoR-40) scores were collected from both groups.

**Results:**

No statistically significant differences were observed in baseline demographic and clinical characteristics between the two groups. At 24 h postoperatively, patients in the TEAS group demonstrated significantly higher QoR-40 scores and significantly lower NRS pain scores and postoperative SAI scores compared to the control group.

**Conclusion:**

TEAS was effective in reducing postoperative pain and anxiety levels while enhancing the quality of postoperative recovery in patients undergoing pterygium surgery.

**Clinical trial registration:**

https://www.chictr.org.cn, identifier ChiCTR2200056062.

## Introduction

Pterygium is recognized as one of the most common ocular surface diseases, typically presenting with clinical symptoms such as ocular hyperemia and vision loss (1). Epidemiological data from 2017 indicated that the overall prevalence of pterygium in China reached 9.9%, while the global prevalence was approximately 10.2%, with a notably higher incidence observed among older adults (2).

Pterygium excision combined with keratoplasty currently represents the preferred surgical approach to treat pterygium and reduce recurrence rates (3). However, conjunctival and corneal trauma resulting from pterygium excision, as well as postoperative suture irritation, frequently lead to ocular discomfort. Approximately 60% of patients experience significant postoperative pain and other irritation symptoms following surgery (4). Pain management strategies commonly involve psychological interventions and the administration of analgesic medications. Nevertheless, simple psychological interventions often fail to provide sufficient pain relief, while analgesic use may result in adverse effects, including nausea, vomiting, and withdrawal syndromes (5). Additionally, uncertainty regarding surgical efficacy and concerns about postoperative recurrence frequently trigger anxiety and fear, especially among older adults. Older adults face an increased risk of complications related to anesthesia and surgery when intraoperative analgesics are administered (5). Therefore, identifying an effective intraoperative intervention to alleviate pain and anxiety in older adults remains crucial.

These challenges collectively impact early postoperative recovery in older adults. The concept of enhanced recovery after surgery (ERAS), proposed and promoted in 1997 (6), emphasizes the importance of effective multidisciplinary communication and collaboration to enhance treatment efficiency and accelerate recovery. Transcutaneous electrical acupoint stimulation (TEAS) is a non-invasive technique involving the application of electrode pads to the skin over specific acupoints. A growing body of research indicates that TEAS can effectively reduce postoperative pain and anxiety while facilitating improved recovery outcomes (7–9).

The primary objective of the present study was to investigate the effect of TEAS applied to bilateral LI4 and PC6 acupoints on the quality of postoperative recovery in older adults undergoing pterygium excision. The secondary objective was to evaluate postoperative pain and anxiety during the surgical procedure in this patient population.

## Materials and methods

### Participants and selection criteria

Based on the results of the previous pilot study, the QoR-40 scale score was set as the primary outcome measure. The mean QoR-40 scale score of the experimental group was 186.4 ± 4.11, and that of the control group was 183.4 ± 4.90. With *α* = 0.05 (two-sided) and *β* = 0.10, the sample size for each of the test group and control group was calculated to be 49 cases using PASS 15.0 software. Assuming a 10% loss to follow-up rate for the study subjects, the final sample size for each of the test group and control group was calculated to be 55 cases, with a total sample size of 110 cases.

A total of 110 older adults who underwent elective unilateral pterygium excision at the First Hospital of Wenzhou Medical University between February 2022 and August 2022 were recruited for this study. This study was based on the completely random grouping method using a computer. Using Stata 15.0 software (StataCorp LLC, College Station, TX, United States), the runiform function was applied to divide the participants into the intervention group and the control group in a 1:1 ratio (with 55 participants in each group). The random grouping results were placed in sealed, sequentially numbered opaque envelopes. Anesthesiologists would open the numbered envelopes in sequence to obtain the random allocation results. Researchers, ophthalmologists, and the patients themselves who collected the intraoperative and postoperative data were not aware of the grouping situation, but the data analysts were not blinded.

The inclusion criteria were: (1) a clear diagnosis necessitating unilateral pterygium excision; (2) age between 65 and 85 years; (3) normal comprehension and communication abilities; and (4) voluntary participation with a signed informed consent form.

**Figure 1:**
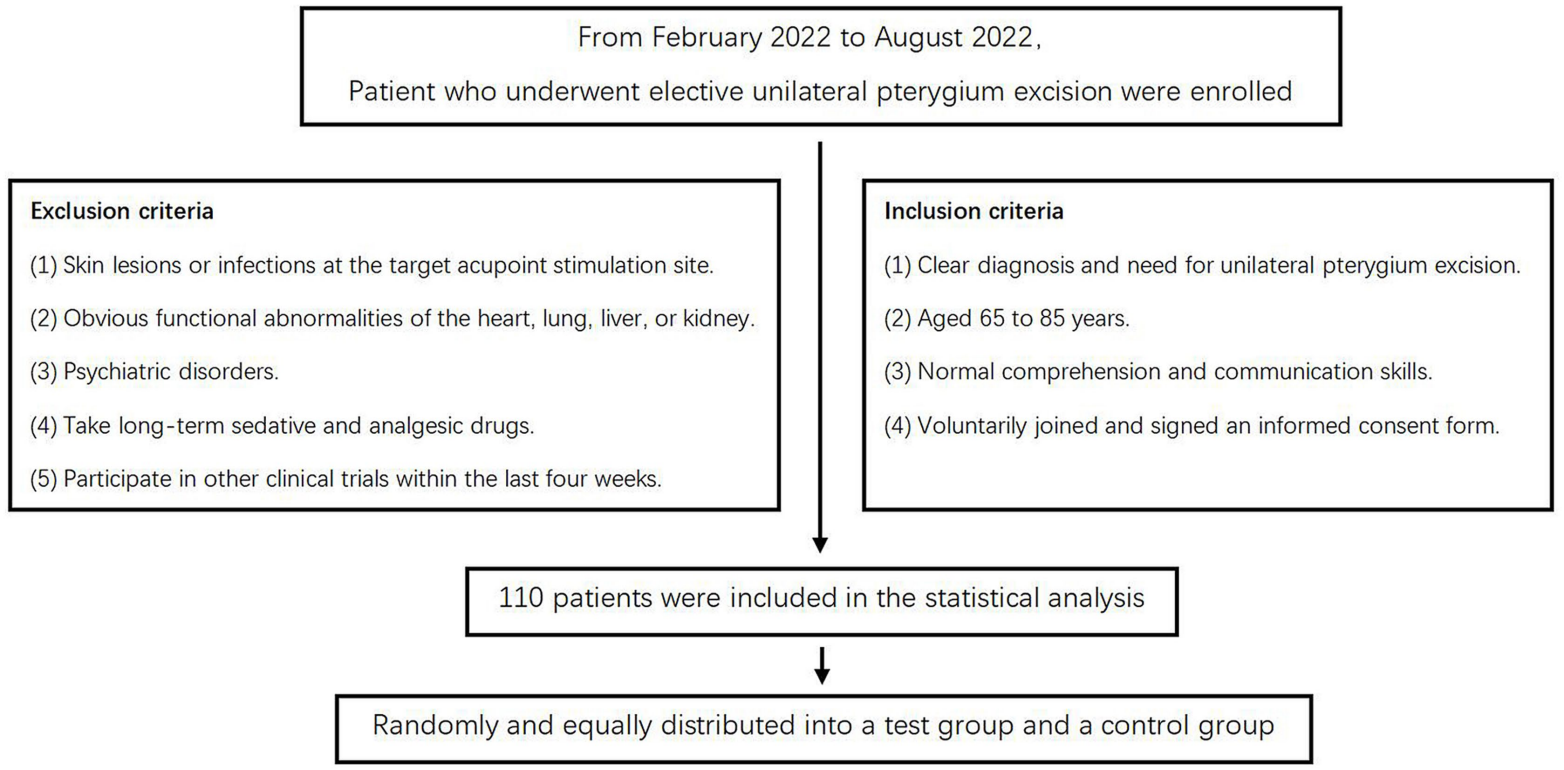
Flow diagram showing selection of study subjects.

The exclusion criteria were as follows: (1) presence of skin lesions or infections at the target acupoint stimulation sites; (2) significant functional abnormalities of the heart, lung, liver, or kidney; (3) diagnosis of psychiatric disorders; (4) long-term use of sedative or analgesic medications; and (5) participation in other clinical trials within the preceding 4 weeks A flowchart demonstrating the participant selection process is presented in Figure 1.

The study protocol was approved by the Clinical Research Trial Record Management Center of the First Affiliated Hospital of Wenzhou Medical University (approval number: KY2021-169) and registered with the Chinese Clinical Trial Registry (registration number: ChiCTR2200056062). Informed consent was obtained from all participants prior to enrollment.

### Standardized surgery and nursing interventions in ERAS

All older adults were admitted to the hospital and received uniform standardized nursing interventions based on the ERAS protocol. Preoperative visits were conducted to assess patients’ conditions and their understanding of the disease, followed by the provision of individualized health education. Patients were informed about the causes, treatment options, and prognosis associated with pterygium, aiming to enhance treatment compliance.

Prior to being transferred to the operating room, ward nurses prepared the patients by cleaning the operated eye, flushing the lacrimal duct, and covering the eye with gauze.

Upon entering the operating room, patients were positioned in a flat supine posture with the head secured using a pillow ring. Fixation training was performed by initially covering the healthy eye. A researcher then positioned a finger approximately 15 cm above the operated eye and slowly moved it downward, guiding the patient’s gaze to follow and then maintain fixation at a designated point for approximately 1 min (10, 11).

The surgical procedure began with routine disinfection and draping of the operative field. Local infiltration anesthesia was administered via subconjunctival injection of lidocaine. The conjunctiva was incised at the neck of the pterygium and separated outward from the pterygium tissue. In cases of significant bleeding, compression using a cotton ball was employed. After complete removal of the pterygium, a conjunctival flap of appropriate size was created at the corneal limbus. The excised conjunctival flap was positioned over the exposed sclera, with its corneal margin aligned to the edge of the exposed area, and sutured to the superficial sclera using a 10–0 nonabsorbable suture. Upon completion of suturing, the surgical field was irrigated thoroughly with saline to eliminate any residual tissue. Tobramycin dexamethasone ophthalmic ointment was applied to the ocular surface of the operated eye, and a gauze dressing was placed.

Following surgery, patients were returned to the ward, where the condition of the dressing on the operated eye was closely monitored. Dressings were promptly replaced in cases of abnormal exudation. Postoperative pain management was emphasized, and analgesics were administered as prescribed when patients reported pain. The pterygium surgery in our hospital is a day procedure. The data can be collected more conveniently 24 h after the operation.

### Intervention

**Figure 2:**
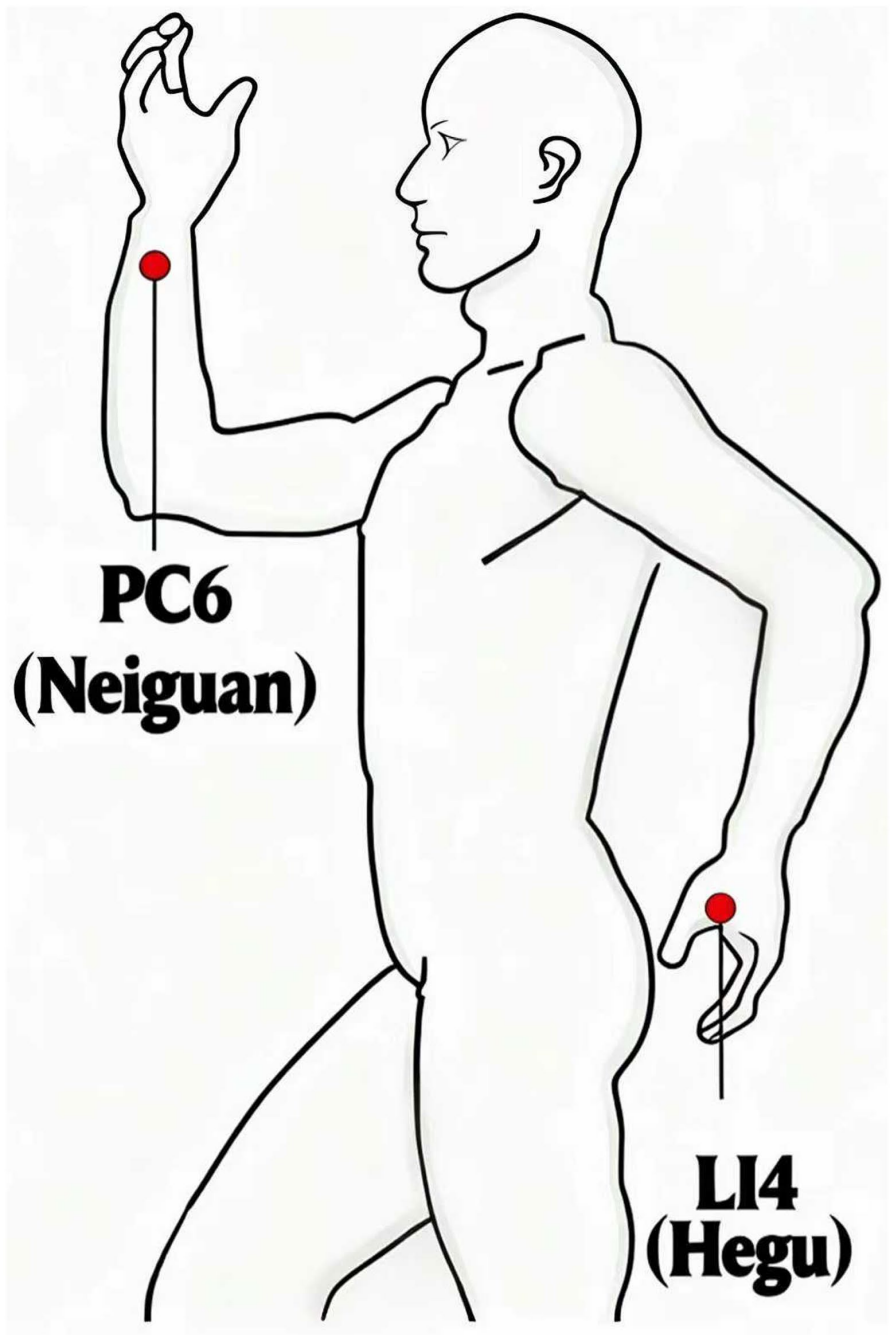
Illustration of the acupoints. PC6 is two inches above the wrist stripe, between the long tendon of the palm and the flexor tendon of the wrist of the radial side. L14 is located in the back of the hand, between the first and second metacarpals, the second metacarpal bone radialis midpoint.

The HANS-200 transcutaneous acupoint nerve stimulator (lot number: 200110514089) was utilized for acupoint stimulation. The skin over the bilateral LI4 and PC6 acupoints (as shown in Figure 2) was cleaned, and electrode pads were attached. Patients in the TEAS group received low-frequency pulsed current stimulation with a 2/100 Hz dense-disperse wave beginning 30 min prior to the start of surgery and continuing until the end of the procedure. The stimulation intensity was gradually increased from weak to strong until the maximum level tolerated by each patient was reached (4–12 mA). In the control group, patients were connected to the HANS-200 stimulator; however, no electrical stimulation was applied (12). The TEAS stimulators for the two groups of patients were of the same model and had the same appearance. The screen parameters were masked, and the patients were not informed about what sensations they would experience from the TEAS, in order to reduce the psychological influence on the patients.

### Measurements

General demographic and clinical information collected included age, gender, height, weight, body mass index (BMI), education level, and surgical history.

Clinical indicators were assessed, including trait anxiety scores, vital signs (heart rate and mean arterial pressure) recorded before surgery, during surgery, and at the end of surgery, as well as intraoperative and postoperative analgesic use.

The primary objective of the study was to assess the quality of recovery using the 40-item Quality of Recovery questionnaire (QoR-40) at 24 h postoperatively.

The secondary objectives included the evaluation of pain using the Numerical Rating Scale (NRS) at the end of surgery, and at 3 and 24 h postoperatively, as well as assessment of anxiety using the State–Trait Anxiety Inventory (S-TAI) at 10 min before surgery and at the end of surgery.

### Statistical analysis

Statistical analyses were conducted using SPSS version 25.0. Parametric data are expressed as mean ± standard deviation (SD) and comparisons between groups were performed using the Student’s *t*-test. Non-parametric data were compared using the rank sum test. Categorical variables were analyzed using the chi-squared test. A value of *p* < 0.05 was considered to indicate statistical significance.

## Results

### Patient characteristics

No statistically significant differences were observed in baseline demographic and clinical information between the two groups (*p* > 0.05). Variables including age, height, weight, BMI, male-to-female ratio, duration of surgery, history of surgery, health insurance participation, and education level were similar and comparable between the TEAS group and the control group (Table 1).

**Table 1:** Demographic and clinical characteristics of the patients (x_±s).

Group		TEAS group (*n* = 55)	Control group (*n* = 55)	*p*-value
Age (years)		70.1 ± 1 0.1	71.5 ± 8.8	0.435
Height (cm)		161.6 ± 7.0	161.7 ± 7.2	0.925
Weight (kg)		62.1 ± 8.2	61.7 ± 9.1	0.804
BMI (kg/m^2^)		23.8 ± 2.6	23.5 ± 2.7	0.609
Duration of surgery (min)		36.8 ± 12.0	36.0 ± 11.2	0.716
History of surgery	Yes/no	33/22	31/24	0.699
Gender	Male/female	32/23	33/22	0.846
Medical insurance	Yes/no	39/16	38/17	0.835
Education level	Illiterate	24	27	0.977
	Primary school	23	21	
	Junior high school	3	2	
	Senior high school	4	4	
	University	1	1	

### Perioperative vital signs

Heart rate and mean arterial pressure measured before surgery, at the beginning of surgery, during surgery, and at the end of surgery demonstrated no statistically significant differences between the two groups (*p* > 0.05) (Table 2).

**Table 2:** The heart rate and mean arterial pressure of patients at each time (x_±s).

Groups	Before surgery	During surgery	At the end of surgery
Heart rate (bpm)			
TEAS group	69.0 ± 6.7	67.1 ± 7.5	67.4 ± 7.8
Control group	69.2 ± 9.3	69.6 ± 8.0	70.76 ± 10.3
*p*-value	0.897	0.085	0.055
Mean arterial pressure (mmHg)			
TEAS group	104.4 ± 13.5	98.6 ± 12.4	98.96 ± 12.6
Control group	104.5 ± 16.0	102.3 ± 14.0	101.4 ± 16.8
*p*-value	0.969	0.148	0.399

### Experimental indexes

#### QoR-40 scores at 24 h postoperatively

**Figure 3:**
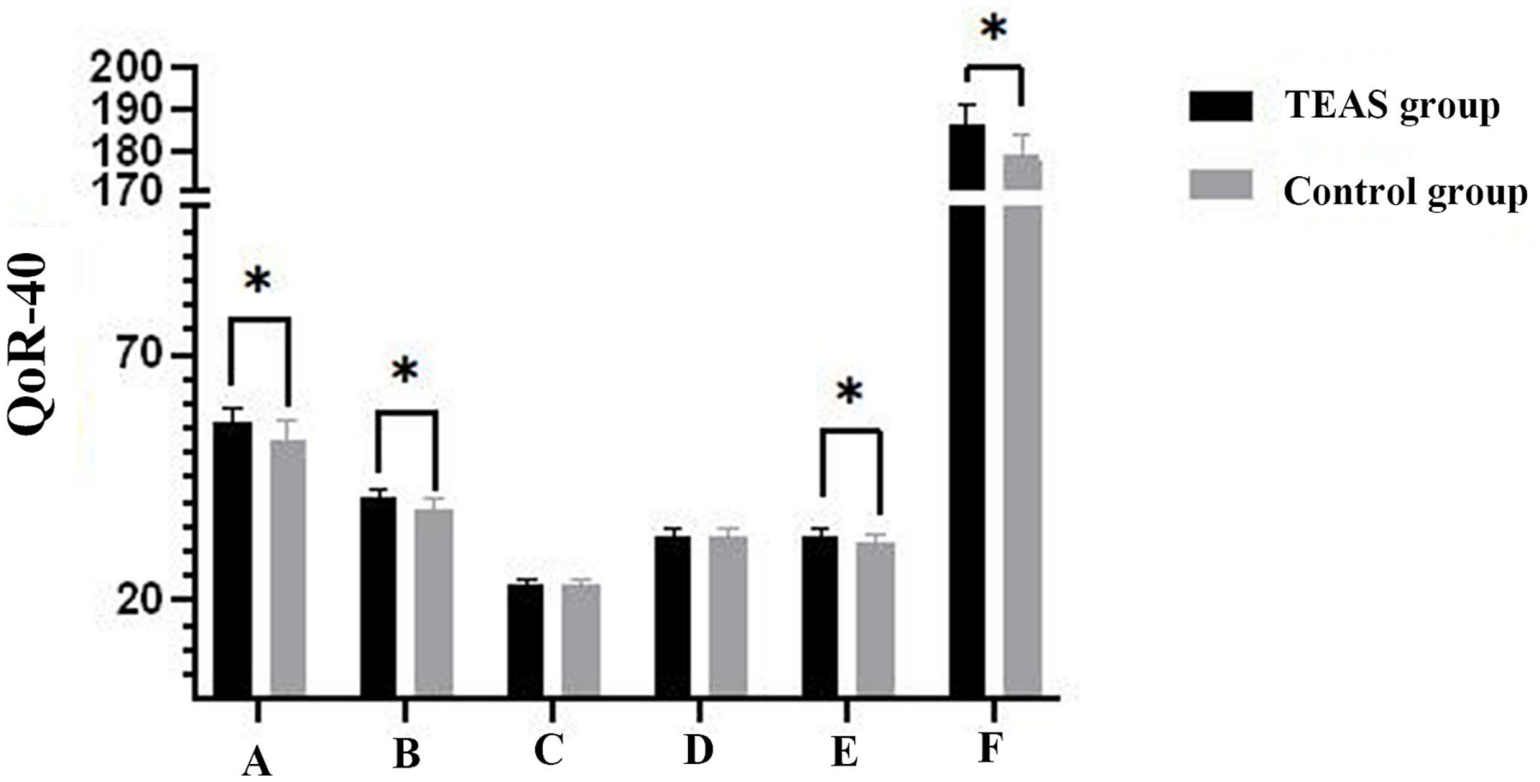
Histogram of the QoR-40 scores at 24 h after surgery. Compared with Control group *p < 0.001. A, comfort; B, emotions; C, physical independence; D, patient suppport; E, pain; F, global QoR-40 score. QoR-40, quality of recovery-40 questionnaire; TEAS, transcutaneous electrical acupoint stimulation.

At 24 h after surgery, patients in the TEAS group exhibited significantly higher QoR-40 scores compared to those in the control group (*p* < 0.05). Specifically, scores related to physical comfort, emotional state, and pain were significantly higher in the TEAS group (*p* < 0.05), whereas differences in psychological support and self-care ability scores were not statistically significant between the groups (*p* > 0.05) (Table 3; Figure 3).

**Table 3:** Comparison of QoR-40 dimension scores at 24 h after surgery between the TEAS and control groups.

Group	Comfort	Emotions	Physical independence	Patient suppport	Pain	Global QoR-40 score
Test group	56.11 **±** 3.02	40.80 ± 2.00	23.02 ± 1.31	33.27 ± 1.39	33.15 ± 1.56	186.35 ± 4.54
Control group	52.47 ± 4.16	38.55 ± 2.19	23.24 ± 1.19	33.11 ± 1.54	31.56 ± 1.75	178.93 ± 5.30
*p*-values	<0.001	<0.001	0.362	0.56	<0.001	<0.001

#### Pain numerical rating scale

**Figure 4:**
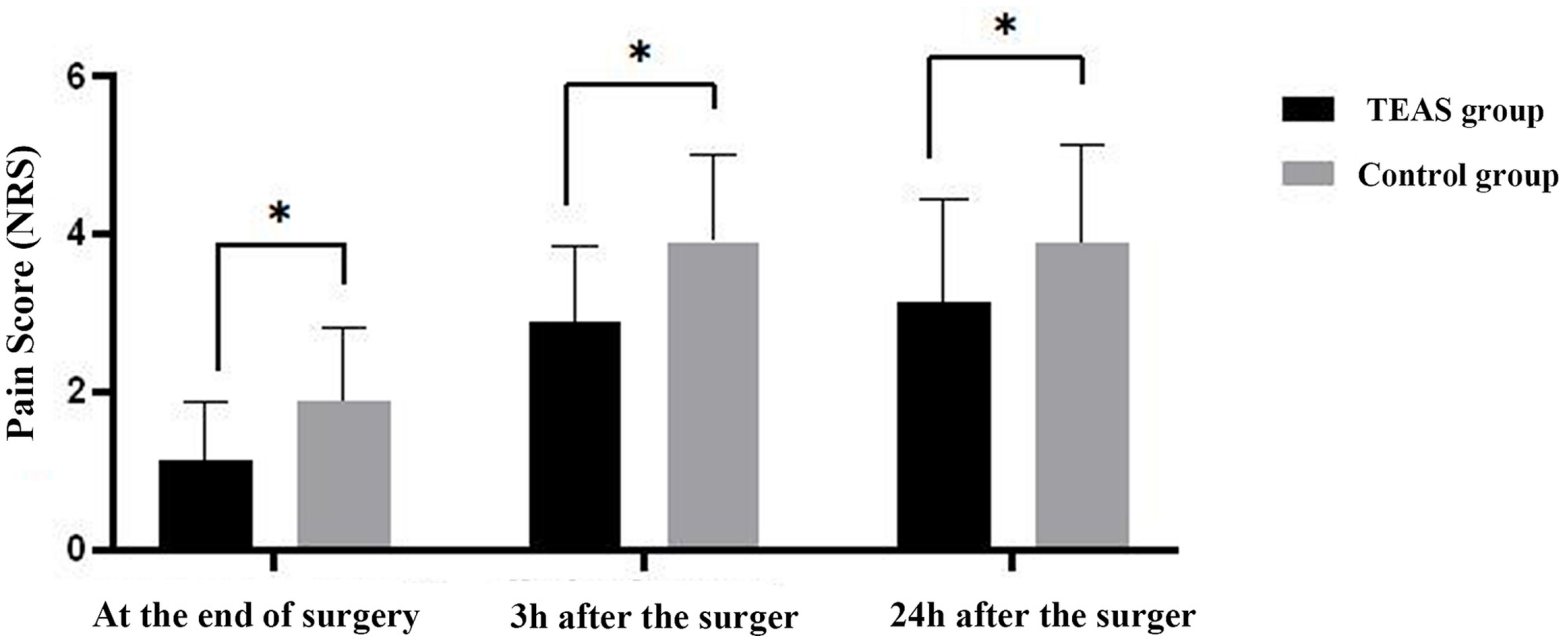
Histogram of anxiety NRS score of two groups at each time. Compared with Control group, *p < 0.05. NRS, Numerical Rating Scale; TEAS, transcutaneous electrical acupoint stimulation.

Pain scores measured using the NRS were significantly lower in the TEAS group compared to the control group at the end of surgery, 3 h postoperatively, and 24 h postoperatively (*p* < 0.05), indicating that TEAS exerted a beneficial analgesic effect following pterygium excision (Figure 4).

#### Intraoperative and postoperative analgesic use

The intraoperative use of proxymetacaine hydrochloride and the postoperative use of somedon were lower in the TEAS group compared to the control group; however, these differences were not statistically significant (*p* > 0.05) (Table 4).

**Table 4:** Intraoperative and postoperative analgesic use.

Groups	Proxymetacaine hydrochloride (use/not use)	Somedon (use/not use)
TEAS group	4/51	20/35
Control group	8/47	25/30
*P*-value	0.359	0.332

#### State–trait anxiety inventory scores

**Figure 5:**
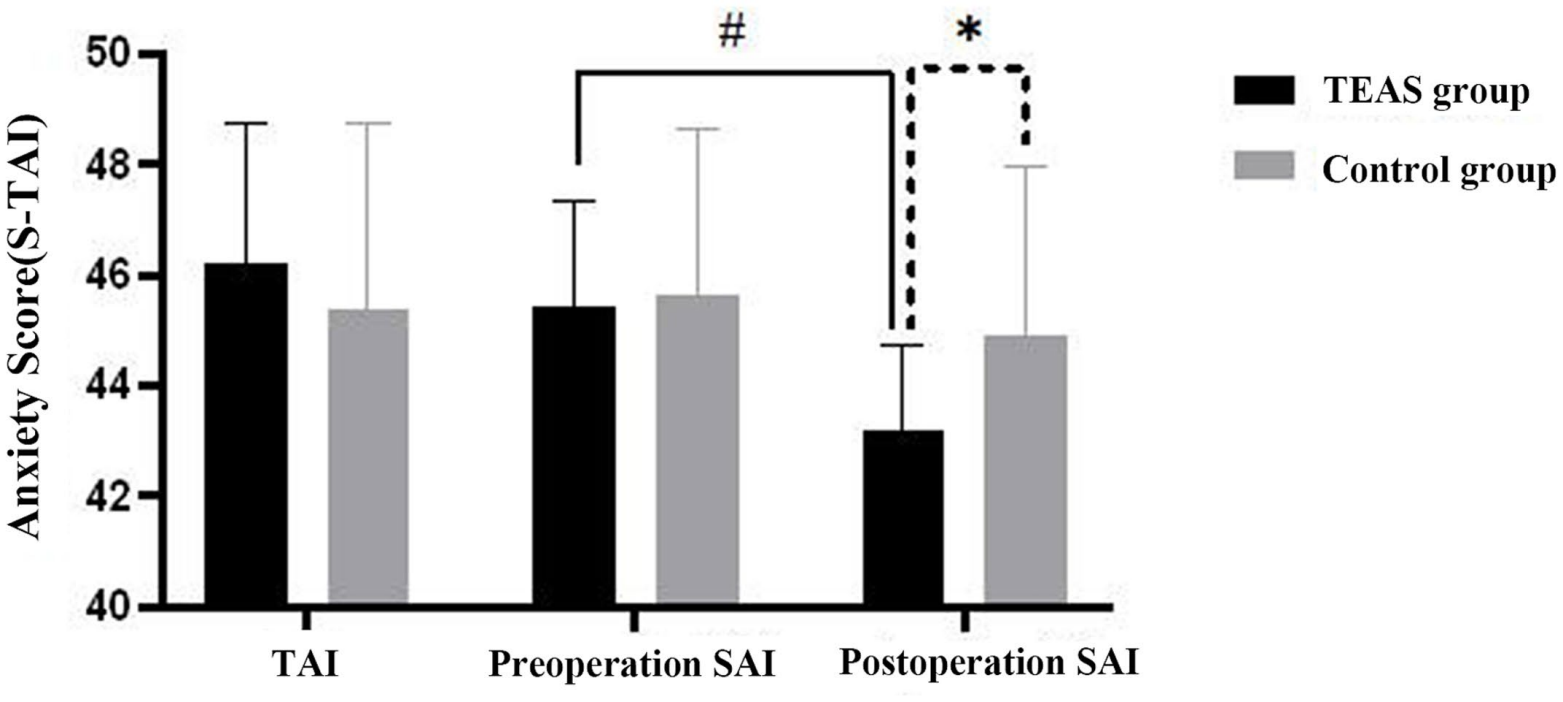
Histogram of anxiety SAI scores. Postoperative SAI scores in the TEAS group compared with the control group at the same time point, ^*^p < 0.001; Comparison of preoperative SAI and postoperative SAI in TEAS group, ^#^*p* < 0.001. TAI, Trait Anxiety Inventory; SAI, State Anxiety Inventory; TEAS, transcutaneous electrical acupoint stimulation.

The S-TAI, consisting of the State Anxiety Inventory (SAI) and the Trait Anxiety Inventory (TAI), was assessed. Postoperative SAI scores were significantly lower in the TEAS group compared to the control group (*p* < 0.05). Furthermore, postoperative SAI scores within the TEAS group were significantly reduced compared to preoperative SAI scores (*p* < 0.05) (Figure 5).

## Discussion

Local anesthesia is the primary anesthetic method used in pterygium surgery (13). However, patients who remain awake during surgery often experience heightened anxiety, which can lead to various detrimental effects, particularly in older adults. Anxiety in these patients can cause perioperative hemodynamic instability and increase the risk of cardiovascular and cerebrovascular events. Therefore, it is crucial to evaluate and mitigate anxiety levels in older adults.

The cornea contains a high concentration of nerve terminals, making postoperative pain after pterygium excision a common experience. Patients often report difficulty in opening the operated eye, excessive tearing, and the sensation of a foreign body in the eye following surgery (14). Various methods have been explored to manage these symptoms, including ice-packing, tight bandage patching, and therapeutic contact lenses. However, these approaches have limitations. Tight bandage patching, while commonly used, has drawbacks such as an unsightly appearance, impaired vision, and discomfort, including sensations of pressure and disrupted blinking in the operated eye. The effectiveness of therapeutic contact lenses in managing symptoms, particularly concerning their impact on the tear film and the patients’ subjective experience, remains inconclusive. Daglioglu et al. reported that soft contact lenses reduced pain within 48 h postoperatively (15). In contrast, Prat et al. and Yeung et al. concluded that therapeutic contact lenses did not alleviate postoperative pain (16, 17). Additionally, the use of analgesics for pain relief is not without its risks. The cornea has relatively fewer blood vessels, making intramuscular or intravenous analgesics less effective. Moreover, the administration of pain medications may result in adverse effects, further complicating pain management.

TEAS, a novel form of acupuncture therapy, offers several advantages, including being non-invasive, free from toxic side effects, and exerting minimal physiological interference. In the context of the evolving concept of ERAS, TEAS has demonstrated its potential to reduce pain and alleviate anxiety in older adults undergoing pterygium excision. Additionally, TEAS contributes to enhanced patient comfort, increased satisfaction, reduced use of anesthetic medications, and promotes faster recovery after surgery, indicating a promising future for its clinical application.

The L14 and PC6 acupoints were selected for TEAS treatment in this study. The L14 point is renowned for its calming, pain-relieving, and meridian-activating effects on orofacial diseases, as described in the Sizongxuege, a classical collection of verses on acupuncture (18). The PC6 point, according to the Zhenjiu Jiayi Jing (a traditional Chinese medicine text on acupuncture and moxibustion), is associated with the treatment of panic and is known for its effects in calming the mind, regulating qi, and alleviating pain (19). Pan et al. performed TEAS therapy on patients undergoing shoulder arthroscopic surgery at the L14 and PC6 points on the affected side. Their findings indicated a significant delay in the need for additional postoperative analgesia, with a reduced dosage of sufentanil and fewer activations of the patient-controlled intravenous analgesia (PCIA) pump within 24 h post-surgery, compared to the control group (20). Additionally, these two acupoints are easily located and have minimal impact on the surgical procedure, making them convenient choices for clinical application.

In recent years, Kolcaba’s theory of comfort has garnered increasing attention from medical professionals, emphasizing the need for improved quality in postoperative recovery. The QoR-40 questionnaire is the most widely utilized scale in clinical settings and has been demonstrated to be one of the most straightforward, reliable, and effective tools for assessing the quality of early postoperative rehabilitation (21). As a result, the QoR-40 was employed as the primary outcome measure in this study to evaluate the effects of TEAS. In this study, 24-h postoperative QoR-40 scores were collected and analyzed, as it has been established that postoperative discomfort following pterygium excision typically manifests within the first 24 h. The results indicated that the TEAS group achieved higher scores than the control group, indicating that TEAS significantly enhances physical comfort, mental wellbeing, postoperative pain management, and overall quality of recovery in older adults.

Several scientific studies have indicated that TEAS can alleviate pain, reduce the incidence of immunosuppression and postoperative adverse reactions, maintain hemodynamic stability, and protect vital organs, thereby enhancing the overall recovery quality of patients (22, 23). In this study, the secondary objective was to evaluate pain levels using the NRS at three time points: at the end of surgery, 3 h post-surgery, and 24 h post-surgery. The results indicated that the TEAS group exhibited significantly lower NRS scores at all three time points compared to the control group, indicating that TEAS treatment provided effective analgesia for patients undergoing pterygium excision.

In this study, the SAI was used as a secondary measure to assess the levels of anxiety in patients both preoperatively and postoperatively. The sedative effects of TEAS have been observed in various fields of medicine. TEAS has been demonstrated to decrease alpha band power and increase delta band power in the electroencephalogram (EEG), reduce the complexity of EEG signals, and significantly lower the overall strength of brain functional connectivity, all of which contribute to a sedative effect (24). Li et al. further confirmed that TEAS at the PC6 acupoint could enhance brain activity in areas such as the left parahippocampal gyrus and the fusiform gyrus, effectively alleviating anxiety after acupuncture at the PC6 point (19).

Previous clinical studies have also demonstrated that TEAS at L14 and PC6 during radical thyroidectomy and at PC6 and Zusanli during laparoscopic cholecystectomy helped to reduce preoperative anxiety (25, 26). The results of this study indicated that the postoperative SAI score in the TEAS group was significantly lower than that in the control group, indicating that TEAS at the L14 and PC6 acupoints may effectively reduce postoperative anxiety in older adults.

This study has several limitations: 1. This was a single-center study, and additional centers with larger sample sizes are necessary to conduct a higher-quality randomized controlled trial. 2. The size of the pterygium was not controlled as a variable in both groups; however, bias was minimized by strict randomization and by indirectly controlling for it through the collection and analysis of the length of surgery. This was easy to perform but lacked rigor. 3. The TEAS parameters were set based on prior clinical studies. Since there is no standardized normative criteria at present, there is an urgent need for large-scale, multi-center clinical studies to provide data and technical support. Future studies should focus on more systematic and comprehensive investigations into important parameters, such as the selection of acupoints, timing, and duration of electrical stimulation. 4. The follow-up period was too short, only considering the peak period of discomfort in patients with pterygium (24 h after surgery), and no discussion was made on the long-term effects.

## Conclusion

The results of this study indicate that the application of TEAS during the perioperative period for older adults undergoing pterygium excision significantly reduces anxiety, alleviates postoperative pain, enhances patient comfort, and improves the quality of recovery following surgery.

## Data Availability

The original contributions presented in the study are included in the article/supplementary material, further inquiries can be directed to the corresponding authors.
